# Supplementary material for: Are carbon emissions trading and green financial instruments synergistic? -Comprehensive quantitative research based on content analysis
Source: PLoS One. 2024 Mar 7;19(3):e0298601. doi: 10.1371/journal.pone.0298601 (PMC10919601; doi:10.1371/journal.pone.0298601)
Supplement: S1 Data — (ZIP) [file pone.0298601.s001.zip › Data/地级市Z_F工作报告.zip!/║╙▒▒/║¬╡a╩╨/└┤╫╘╣1⁄2╓┌║┼í╛┬φ┐╦╩2╛▌═°í┐_2015.docx]

各位代表：

现在，我代表市人民政府向大会作工作报告，请予审议，并请市政协委员和列席会议的同志提出意见。

一、2014年工作回顾

刚刚过去的2014年，面对复杂严峻的经济形势，市政府在省委、省政府和中共邯郸市委的坚强领导下，在市人大、市政协的监督支持下，紧紧围绕建设宜居宜业宜游富强邯郸、美丽邯郸的战略目标，突出项目、环境、城乡、平安“四大建设”，统筹做好稳增长、调结构、促改革、提效益、惠民生各项工作，攻坚克难，砥砺奋进，较好完成了市十四届人大二次会议确定的工作任务。

（一）积极应对下行压力，经济运行稳中有进。全市生产总值达3080亿元，增长6.5%；一般公共预算收入183.2亿元，可比增长8.2%；固定资产投资3090.7亿元，增长16.1%；社会消费品零售总额1228.9亿元，增长12.3%;出口总值16.6亿美元，增长21.2%；实际利用外资9.3亿美元，增长4.7%。主要经济指标增速达到全省平均水平。在保持经济平稳增长的基础上，发展后劲持续增强，质量效益稳步提高。坚持“三集中”工作法推进重点项目建设，成安友发制管、广平格士高电器、曲周鸿力轴承、涉县视华液晶屏等一批产业项目开工建设或竣工投产。邯长邯济铁路扩能改造、邯大高速、京港澳高速改扩建邯郸段、南水北调邯郸段等一批重大基础设施竣工投用，实现县县通高速公路。加大企业帮扶力度，规模以上工业利润预计增长17.2%，新增入统企业244家。新设立市场主体7.6万户，增量同比增长97.2%，增速全省最高。预计城乡居民人均可支配收入分别达22700元和10340元，分别增长9%和11%，高于生产总值增速2.5个和4.5个百分点。

（二）深入推进结构调整，转型升级步伐加快。“有中生新”改造提升传统产业，钢铁产业整合重组进入实施阶段，冀南钢铁、太行钢铁退城进园升级改造项目签订协议，压减生铁产能390万吨、粗钢产能263万吨。三一玺尧煤制油项目量产出油，煤化工产业链条明显拉长。“三年千项”工程完美收官，累计实施项目1116个、完成投资2620亿元。“无中生有”培育战略性新兴产业，新材料、新能源、家电等产业发展迅速，规模以上装备制造业、高新技术产业增加值分别增长12.1%、21.9%，高于规模以上工业增速7个、16.8个百分点。大力发展现代服务业，制定实施了服务业发展规划，服务业增加值增长9.4%，占生产总值比重提高1个百分点。接待游客和旅游总收入分别增长18.4%和33.8%，临漳邺城公园、魏县梨乡水城被评为国家水利风景区。提高科技创新能力，现代装备制造、煤化工创新型产业集群分别列入国家级、省级试点，新增国家高新技术企业26家、省级工程技术研究中心和工程实验室5家，科技型中小企业达1916家、总量居全省第一。

（三）大力发展现代农业，农村经济稳步发展。粮食总产544.9万吨，连续三年荣获全国粮食生产先进市，蔬菜和肉蛋奶产量稳定增长。积极发展农业产业化经营，新增省级重点龙头企业16家、累计达62家，总量和增量均居全省第一，发放“金财通”贷款6.88亿元，预计产业化经营率达65.3%。华裕家禽、天赐调料荣获中国驰名商标。土地流转面积达208万亩，流转率23.4%，提高4.6个百分点，全省首家农村产权交易市场在邱县成立。地下水超采综合治理进展顺利，完成74万亩综合治理任务。

（四）着力加强污染治理，生态环境日益改善。坚持削煤、改企、降尘、控车、增绿多管齐下，综合整治大气污染，关停取缔重污染企业693家，完成“五个行业”节能减排治理项目306项，淘汰黄标车3.8万辆，拆除锅炉955台、烟囱1650根。削减煤炭消费量431万吨。空气质量达标天数88天，同比增加34天，空气质量综合指数、细颗粒物（PM2.5）平均浓度分别下降23.2%、16.5%。实施山水林田湖整体修复，治理矿山3000多亩，完成造林58.1万亩，森林覆盖率达26.2%。狠抓节能减排，我市列入国家循环经济试点示范市、全国绿色循环低碳交通运输示范市。预计单位生产总值能耗下降5%，提前完成“十二五”节能任务。

（五）统筹城乡协调发展，新型城镇化水平提升。优化中心城市空间结构，部分县区行政区划调整稳步实施。开工市县重点城建工程523项，完成投资631.8亿元。全面推进东区开发建设，“两违”整治成效明显，村庄征迁、路网管网建设加快，科技中心、青少年活动中心等一批重大工程开工建设。加快改造提升老城区，回迁房开工5791套（户）、竣工10721套（户），整治小街巷98条，滏阳河水上旅游向社会开放。新建过街天桥6座，实施邯峰长输供水管线改造，市政设施进一步完善。大力发展公共交通，200部电动公交车分批投用。县城建设步伐加快，19个镇列入全国重点镇。出台城市精细化管理办法，六大专项整治深入开展。实施农村面貌改造提升行动，改厕41万座，393个重点村生产生活条件明显改善。

（六）全面深化改革开放，发展活力持续增强。推进重点领域改革，衔接落实国务院、省政府取消下放调整行政审批事项112项，市本级下放行政审批事项44项，向城区下放经济管理权限，调整了工商、质监行政管理体制。市属国企改革三年目标基本完成，县级公立医院综合改革实现全覆盖。加快对外开放步伐，抢抓京津冀协同发展战略机遇，新兴凌云碳酸氢钠项目成为首个央企整体外迁工业项目，冀津循环经济示范区获批建设，北京大学邯郸创新研究院挂牌成立。与中电投、国机集团等12家央企签订战略合作协议，捷克飞马特、韩国东洋物产等一批跨国企业入驻邯郸。冀南新区获批省级高新技术产业开发区，新增省级经济开发区6家，实现县县有省级开发区。推动金融创新，华夏银行、沧州银行入驻我市，邯郸银行新设市外机构9家，新增挂牌企业15家，成功发行首支中小企业集合债券。全市新增贷款269亿元，同比增长8.9%。大力发展民营经济，预计民营经济增加值增长7%以上，我市入围中国企业500强5家、民营企业500强10家、河北百强企业26家，入围企业个数均居全省第一。

（七）切实保障改善民生，社会发展和谐稳定。实施十大民生工程，96件实事如期完成，财政用于民生支出239.2亿元,增长3.9%。城镇新增就业10.3万人，农村劳动力向非农产业转移5.4万人。城乡居民社会养老保险参保率达95%，企业退休人员基本养老金人均月增202元，城乡居民低保标准分别增长30.5%和18.3%，新建农村幸福院和社区居家养老服务中心328个，农村“五保”集中供养能力达61%。新农合参合率达99.3%，居全省第一。保障性安居工程开工16537套（户）、竣工28072套（户）、分配入住28913套（户），超额完成省定任务。全市消除了饮用水氟超标问题，基本实现农村安全饮水全覆盖。15万人实现稳定脱贫，肥乡、鸡泽、广平3个县提前脱贫出列。全面发展社会事业，大力推进学前教育三年行动计划和义务教育均衡发展，实施教育质量三年提升工程，建成农村标准化中小学50所。成功举办第十二届国际太极拳运动大会、第九届中原民间艺术节、第二届中国吹歌节，加强赵王城、赵王陵等文化遗产保护开发，大名县列入省历史文化名城，永年县荣获全国文化先进县，新增国家级非物质文化遗产2项，7部作品荣获省“五个一工程”奖，市图书馆新馆对社会开放。围绕培育和践行社会主义核心价值观，深入开展文明城市创建活动，市民文明素质不断提升。圆满完成第三次经济普查工作。创新社会治理，食品药品安全监管不断强化，安全生产形势稳定，信访工作得到加强，医患纠纷大幅减少，着力解决建设领域“两拖欠”，及时处理非法集资问题，深入推进平安邯郸建设，警网、天网、民网“三网合一”防控体系日益完善，刑事案件下降36.1%，其中“两抢一盗”案件下降45.5%，群众安全感和满意度进一步提高。

（八）加强政府自身建设，工作作风明显转变。深入开展第二批党的群众路线教育实践活动，认真落实中央“八项规定”精神，解决了一批群众反映强烈的突出问题。推出34项便民利民措施，实行主要路口“借道左转”和小学弹性离校，设立乡村流动婚姻登记服务站，减少离退休职工领取养老金资格认证次数，在农村设置刷卡机（POS机），参加养老保险的群众实现“缴费不出村、领钱在家门”。加快政府职能转变，在全省率先公开行政权力清单，政府机关标准化管理全面实施。坚持依法行政，成立市政府法律顾问委员会，完成政府规章立法项目5件，自觉接受人大、政协和新闻媒体监督，办理人大代表建议298件、政协提案526件，按时办复率均为100%。加强廉政建设，加大审计监察工作力度，市本级“三公经费”支出下降13%。

同时，国防动员、计划生育、民族宗教、广电出版、外事侨务、邮政通信、妇女儿童、史志档案、防震减灾、人民防空、统计、物价、气象、援疆、老龄、残疾人等工作，都取得了新的成绩。

各位代表！过去的一年，发展困难比预料的多，工作成效比预想的好，成绩来之不易。这是省委、省政府和中共邯郸市委正确领导的结果，是市人大、市政协监督支持的结果，是全市上下拼搏实干的结果。在此，我代表市政府，向全市人民，向人大代表、政协委员，向各民主党派、工商联、无党派人士和人民团体，向驻邯人民解放军、武警官兵和政法干警，向中直、省直驻邯单位，向各位老领导，向所有关心支持邯郸发展的各界人士，表示衷心的感谢！

在看到成绩的同时，我们也清醒地认识到，受支柱行业市场低迷、化解过剩产能、防治大气污染等因素影响，去年全市生产总值、一般公共预算收入等指标没有达到预期目标。经济社会发展还存在不少困难和问题，主要是：产业结构依然偏重，战略性新兴产业、现代服务业支撑作用还不强；产业层次低，创新能力弱，部分企业生产经营困难；化解过剩产能和大气治理压力增大，生态环境治理修复任务艰巨；事关群众切身利益的问题解决得还不够好，一些部门和工作人员办事效率不高，发展环境还需优化。对此，我们一定深入研究分析，认真加以解决。

各位代表！邯郸发展正处于爬陡坡、过深坎的关键时期，虽然形势复杂多变，但机遇大于挑战，利好多于困难。特别是京津冀协同发展和中原经济区两大国家战略的深入实施，带来了千载难逢的历史机遇；一批重大基础设施的建成投用，使邯郸四省交界区域中心城市的地位进一步凸显；省委、省政府大力支持邯郸结构调整和重点区域突破，给予了许多政策支持和要素保障；全市上下风清气正、人心思进，凝聚了奋发有为、干事创业的强大合力。只要我们抢抓新机遇，谋求新作为，就一定能够谱写邯郸转型升级、绿色崛起的新篇章!

二、2015年目标任务和工作重点

今年政府工作的总体要求是：认真贯彻党的十八大和十八届三中、四中全会及中央经济工作会议精神，全面落实省委八届九次全会、全省经济工作会议和市委八届六次全会各项部署，坚持稳步较快增长总基调，保持定力，综合施策，把改革创新和法治建设贯穿经济社会发展的各个领域各个环节，着力提高质量效益，着力促进生态改善，着力保障改善民生，努力开创建设宜居宜业宜游富强邯郸、美丽邯郸的新局面。

全市经济社会发展的主要预期目标是：全市生产总值增长6%左右，一般公共预算收入可比增长5%，固定资产投资增长12%，社会消费品零售总额增长11.5%，进出口总值和实际利用外资均增长5%。城乡居民人均可支配收入分别增长8%和8.5%，居民消费价格涨幅控制在3%左右，城镇登记失业率控制在4%以内，人口自然增长率控制在8.6‰以内。细颗粒物浓度下降6%，单位生产总值能耗和化学需氧量、二氧化硫、氨氮、氮氧化物排放量完成省定目标。

围绕落实上述要求，重点把握好五个方面：一是致力转型发展。主动适应经济发展新常态，统筹发展速度、质量和效益，打好转型升级组合拳，促进长远可持续发展。二是致力创新发展。加快以科技创新为核心的全面创新，坚持需求导向和产业化方向，以科技创新引领转型升级，不断增创发展新优势。三是致力统筹发展。完善新型城镇体系，推进以人为核心的新型城镇化，促进产城互动、融合发展，加快城乡一体化进程。四是致力绿色发展。坚持生态立市，一手抓污染治理，一手抓生态建设，多还生态旧账，不欠环境新账，尽最大努力改善空气质量。五是致力和谐发展。坚持民本为重、民生为先，围绕保基本、补短板、兜底线，加快基本公共服务均等化，保障安全稳定，维护公平正义，让人民共享改革发展成果。

重点做好十个方面工作：

（一）抓项目增投资，持续增强发展后劲。充分发挥投资拉动作用，保持投资强度，优化投资结构，以优质增量扩总量、活存量。

实施重点项目建设“三百工程”。全市抓好100个开工在建、100个竣工投产和100个前期项目，完成年度投资800亿元。全市投资超10亿元的产业项目达到160个，投资增长20%以上。加快推进和争取东郊热电、轨道交通、京涉高速等重大基础设施建设，确保邯郸火车站改扩建、绕城高速东南环竣工投用，实现县县通天然气。

突出重大项目带动。集中支持“双三十”重大项目，抓好中船重工汉光光电科技园等30个重大在建项目，加快推进新峰煤制天然气等30个重大前期项目。把引进带动能力强的大项目作为重中之重，确立“215”目标，每年洽谈和引进2个投资超百亿元的战略支撑项目、15个投资超50亿元的大项目，带动全市转型升级、绿色崛起。

建强项目聚集平台。加大园区投入，完善配套设施，提高全市园区聚集项目水平。努力打造冀南新区、经济技术开发区两大“引爆点”，着力引进投资超百亿元的战略支撑项目，如期实现“三年倍增”目标。突出抓好冀南新区台湾产业园、经济技术开发区欧洲产业园、武安中欧产业园、魏县台资装备产业园等国别（地区）园区建设，提升园区承载能力。

完善项目管理机制。严把项目准入关、投资强度关、产出效益关和环境保护关，加强项目跟踪问效，完善项目考核办法，实施差别化要素配置改革试点工作。将新增土地指标的60%以上用于省市重点项目，研究出台鼓励企业建设多层厂房、节约集约用地的政策措施，提高土地利用率。严格执行项目预警和退出机制，实行土地指标动态管理，对圈而不建的坚决依法收回，切实把土地这一稀有资源用在大项目、好项目上。

（二）抓调整优结构，力促工业转型升级。坚持“有中生新”、“无中生有”两手抓，持续推进产业结构调整，尽快“让老树开出新花、让新芽长成大树”。

打好钢铁整合重组攻坚硬仗。加快冀南钢铁、太行钢铁、永洋特钢整合重组步伐，力争上半年开工建设，着力做好宝信钢铁转型升级等前期工作，完成省下达的化解钢铁过剩产能任务，促进钢铁产业装备水平提升、产品结构优化、环保设施达标、企业效益提高。支持钢铁深加工项目扩规模、上档次，提高钢铁就地转化率。同时，加大企业技术改造力度，推动煤炭、建材、电力等传统产业向中高端迈进。

发展壮大战略性新兴产业。按照“2+5”的思路，加快发展新材料和先进装备制造产业，培育发展生物、电子信息、新能源、节能环保和新能源汽车产业，持续壮大、引进一批龙头项目，培育新的经济增长点。支持现有产业企业对标行业高端，明确主攻方向，尽快做大做强，提高对经济增长的贡献率。

强化工业运行调节。健全工业运行联席会议制度，定期研究解决企业发展难题。加强“个转企、小升规”工作，新增规模以上工业企业200家以上。搞好市场帮扶，本地产品优先列入政府采购目录，鼓励企业同质同价优先采用本地产品。搞好金融帮扶，加强政银企沟通对接，开展“助保贷”、“金桥帮扶”等融资支持，帮助企业渡过难关。

（三）抓三产扩规模，推动现代服务业提速发展。像抓工业一样抓服务业，着力扩规模、优结构、提质量，加快服务业与工农业协同融合发展。年内，引进20个投资超10亿元的现代服务业项目。

繁荣发展商贸物流业。发挥国家二级物流园区布局城市的优势，培育壮大冀中能源国际物流、鼎峰物流、万合集团等龙头企业，突出抓好林安智慧商贸物流城、武安保税物流中心、润恒冷链物流园等现代物流项目，确保国际陆港投入使用，加快推进友谊时代广场等大型商贸综合体项目建设，推动商贸物流业快速发展。实施“农超对接”，提升农副产品在京津市场份额。

做大做强文化旅游业。打造一批精品旅游线路，编制太行山旅游交通线网规划，争取开通“北京西—邯郸”旅游观光列车。提升龙头景区建管水平，完善配套设施，确保娲皇宫成功创建5A级景区。推出“金中原惠民旅游一卡通”，积极开辟韩国市场，力争接待国内外游客人数、旅游总收入分别增长10%和20%以上。

大力培育新兴服务业。适应消费结构升级新趋势，积极培育云计算、服务外包、文化创意、金融租赁等新兴服务业，突出抓好河北网商、恒富电商、平原电商等电子商务园区建设，鼓励引导社会力量发展健康养老、信息消费、教育培训等多种服务业态，促进新兴服务业快发展、大发展。

（四）抓“三农”夯基础，不断提升农业现代化水平。转变农业发展方式，突出抓好“水厕菜奶”等重点工作，让农业更强、农民更富、农村更美。

加快农业产业化步伐。在稳定粮食生产的基础上，按照“抓好龙头、构好机制、建好基地”的思路，重点推进子丰多肽产业园、康诺乳业扩建等100个农业产业化项目建设，扶持壮大30家示范性龙头企业，新增30家销售收入超亿元龙头企业。加快邯郸国家级农业科技园区建设，支持各县（市、区）建设现代农业示范园区，打造一批蔬菜、肉蛋奶优质农产品生产基地。全市农业产业化经营率达到66%以上。

积极稳妥推进土地流转。加快农村土地确权颁证，实现邱县确权登记颁证全覆盖，其它县（市、区）累计完成60%以上。扩大县级农村产权交易市场试点，广平县、峰峰矿区等4个县级农村产权交易市场挂牌运营。整合涉农项目资金，集中扶持家庭农场、种养大户、农民合作社等规模经营主体，年内创建示范性家庭农场200家以上。农村土地流转率达到27%。

深化农村面貌改造提升行动。加强村庄规划建设管理，积极探索符合农村实际的美丽乡村创建模式，充分调动农民群众参与积极性。坚持试点先行，着力打造邯郸县1个省级整体推进县、3个市级片区、28个县级片区，创建100个“小第八村模式”示范村，带动全市农村面貌大提升。

（五）抓城乡重统筹，着力加快新型城镇化进程。发挥城镇化扩内需、稳增长的作用，坚持以人为本，走集约、智能、绿色、低碳的新型城镇化道路。

强化规划引领。进一步完善城乡规划体系，提升规划管理水平，积极开展“多规合一”工作。对城市的重要节点和重点区域，精雕细刻，搞好修建性详规和城市设计。坚持规划即法、执法如山，杜绝盲目决策、随意建设。

实施一批重点城建工程。坚持统筹安排、量力而行、急需为先、保证续建，有序推进重大城建工程。突出抓好东区开发，启动中央商务区建设，加快实施科技中心、青少年活动中心、传媒中心、妇女儿童中心等公共设施项目，完成客运枢纽一期工程。按照建设时序，加快村庄征迁、回迁房建设，推进新区路网、场站、河道等基础设施建设。完善老城区市政设施建设，实施一批民生城建项目，打通城市内道路，优先发展公共交通，缓解交通拥堵问题。加强地下管线建设管理，确保城市防汛排涝安全。强化工程质量监管，创新多元融资和可持续资金保障机制，杜绝“豆腐渣”工程和“半拉子”工程。同时，认真落实支持房地产业发展的意见，促进房地产业平稳健康发展。

加快县城扩容升级。优化详规设计，突出地域特色，围绕生态、文化、产业融合发展，完善公共配套设施，提升综合承载能力。科学布局县城和开发区，合理规划和控制连接过渡地带，实现产城互动。突出重点，因地制宜，培育一批特色小城镇，让农村居民实现就近城镇化。创新户籍管理体制机制，有序推进农业转移人口市民化。全市城镇化率提高0.5个百分点。

加强城市精细化管理。全面加快智慧城市建设，完善通信网络设施，抓好云计算中心、居民一卡通等项目建设，支持丛台区、永年县和其他有条件的县加快信息化步伐，促进信息化与城市发展深度融合。深入推进城市精细化管理，抓好先行试点，以政府购买社会服务的方式，做好环境卫生、园林绿化、市政设施维护等工作。把今年作为城市管理“严管年”、精细化管理“落实年”，突出抓好城市卫生、小街巷、老旧小区物业、停车秩序、建筑围挡等治理，让我们共有的生活家园更加整洁、舒适、宜居。

（六）抓生态治污染，持续改善大气环境质量。坚持以铁的手腕治理大气污染，坚决打赢这场攻坚战，让邯郸的天更蓝、水更净、空气更清新。

抓好重点行业治理。实施煤电节能减排升级改造行动计划，推进煤炭清洁高效利用，全部淘汰市区和各县城区10蒸吨及以下燃煤锅炉。加大工程减排力度，如期完成“五个行业”综合治理工程。严格执行钢铁、电力、水泥、焦化行业大气污染物排放新标准，全面改造不能稳定达标排放的污染治理设施，坚决关停排放不达标的重污染企业。综合整治城市及周边扬尘，大力推广新能源汽车，全部淘汰黄标车，最大限度减少机动车污染排放。

搞好生态整体修复。持续开展“绿美邯郸”攻坚行动，完成造林绿化52.5万亩，全市森林覆盖率达到27.7%以上。治理水土流失面积150平方公里。加快推进黑龙洞泉域水源置换，加强饮用水源地保护。搞好地下水超采综合治理，年内压采地下水7000万立方米。实施引黄入冀补淀相关工程，加快南水北调配套工程建设，上半年完成水厂以上管网工程和19个水厂建设任务。

完善污染防控机制。以新《环保法》实施为契机，加大违法排污处罚力度，推动防治工作长效化、法制化。建立环保、公安联合执法机制，对环境违法犯罪行为露头就打、绝不姑息。注重用经济手段和市场机制加强环境保护，积极探索和推进排污权交易，实施全天候、全过程在线监测，坚决杜绝偷排偷放行为。

（七）抓改革促开放，进一步改善发展环境。把改革作为创新发展的点火器，把开放作为万民创业的活力源，打造有利于先进要素竞相涌入的一流环境。

持续深化重点领域改革。加快行政审批制度改革，落实和承接好国家、省取消下放的行政审批事项，5月底前全面取消非行政许可审批事项，最大限度地简政放权。完善“三级平台”、“两个代办”制度，建立统一规范的公共资源交易平台，6月底前全面建成全市便民服务体系。加快国有企业改革，基本完成县属国有工业企业改革任务。深化工商登记制度改革，积极推行工商营业执照、组织机构代码证和税务登记证“三证合一”登记制度。统筹抓好教育、文化、医疗、价格、事业单位等领域的综合改革。

全方位扩大对外开放。创新招商机制，市、县成立专门招商团队，给任务、给条件、给待遇，提高招商引资实效。发挥4个市级专业招商组的带头作用，尽快开展专业化招商。坚持内资外资项目一起抓，年内引进20个投资超亿美元的外资项目。坚持“引进来”与“走出去”并重，鼓励有条件的企业走出去，开拓国外市场，不断扩大出口规模，提升经济外向度。把外贸工作作为反向推手，鼓励引导企业放眼全球，与国际市场接轨，倒逼企业提高产业层次，生产更多有市场竞争力的终端产品。

下大力优化发展环境。整治企业周边环境，严厉打击强揽工程、阻挠施工等违法行为。全面优化政务环境，集中整顿不作为、慢作为、乱作为和吃拿卡要等突出问题。加大明查暗访力度，对破坏发展环境的案件，发现一起，曝光一起，严惩一起。

（八）抓创新强支撑，增创发展新优势。强化创新思维，壮大创新主体，搞活创新机制，让创新发展成为时代主旋律。

实施协同创新。借势借力京津冀协同发展战略和中原经济区规划实施，最大限度地引进各类创新要素。瞄准北京，对接央企、金融机构和跨国企业总部，全方位对接争跑，在扩大开放、深化合作方面有更大突破。开工建设中科院邯郸科技园，北京大学邯郸创新研究院首批5个高科技项目实现产业化。

加强自主创新。认真落实关于促进科技型中小企业发展的政策措施，确保科技型中小企业新增600家以上，达到2600家。发挥企业主体作用，加大高新技术企业培育力度，争创2家省级创新型企业，力争高新技术企业达到100家以上。支持企业建立研发机构，推动晨光色素工程技术研究中心进入国家序列，加快国家煤化工产品质检中心建设，力争新增12家省级以上企业技术中心。加大招才引智力度，以灵活方式引进一批高层次人才，支持科技人员创办领办科技型企业。推进科技成果转化，加快中船重工718所科研成果军转民步伐，实现成果产业化和资本化。

搞好金融创新。适应财政金融新体制，转变政府融资思路，更多地发挥金融和资本市场作用，用活金融工具，破解资金难题。积极引进外地产业基金机构，鼓励民间资本发起设立创业投资基金和股权投资基金，探索推广政府与社会资本合作（PPP）模式，吸引更多的社会资本投入城市基础设施建设和运营。加强企业上市培育，争取新增6家挂牌企业，通过2—3年努力，使我市上市企业数量达到全省平均水平。引导金融单位积极创新金融产品，扩大融资和贷款规模，支持企业新上项目和扩大再生产。严厉打击恶意逃废金融债务行为，着力优化金融生态环境。

（九）抓县域育特色，提升区域综合竞争力。把县域经济作为全市科学发展、绿色崛起的基石，做大规模、做强产业、做出特色。

统筹区域协调发展。围绕西部提升、中部繁荣、东部振兴，制定县域经济发展推进计划，统筹县域经济发展的目标定位，完善县域经济差异化考核办法，促进县域分工协作，实现错位发展。支持西部县（市、区）改造提升钢铁、煤炭等传统支柱产业，发展壮大战略性新兴产业，进一步强支撑、促转型；支持中部区县发展现代服务业和高新技术产业，进一步提档次、增效益；支持东部县加快新型工业化和农业现代化步伐，进一步扩总量、快发展，形成布局科学、良性互动的发展格局。

壮大特色产业集群。搞好规划引领，优化资源配置，强化龙头带动，着力培育现代装备制造、农业机械、新能源及新能源汽车、新材料、现代煤化工、家电、天然植物提取物等7个创新型产业集群，壮大提升制管、棉纺、标准件、铸造、食品加工、轴承等6个特色产业集群，力争通过3年努力，全市新培育营业收入超500亿元产业集群2个、超100亿元产业集群7个。

放手发展民营经济。坚持法无禁止即可为，鼓励民营企业进入交通、能源、水利、环保、医疗等领域。不折不扣地落实扶持民营经济、支持全民创业的政策措施，健全完善创业辅导、人才培训、技术支持、信息咨询等公共服务体系，最大限度地激发全民创业热情。全市新设立市场主体3.5万户，民营经济增加值占生产总值比重提高1个百分点，实缴税金占全部财政收入比重提高1个百分点。

（十）抓民生铸和谐，不断增进人民福祉。越是经济形势困难，越要更加关注和改善民生，全力做好顺民心、解民难、保民安的各项工作。

全力办好民生实事。财政用于民生投入230亿元以上。拓宽居民增收和就业创业渠道，激发创业活力，推进更高质量的就业，重点抓好高校毕业生、农村转移劳动力、城镇困难人员、退役军人等重点群体就业，做好化解过剩产能失业人员就业工作，全年城镇新增就业10万人左右。完善社会保障体系，加快实施全民参保登记计划，以中小企业和灵活就业人员为重点，扩大社会保险覆盖面。新农合和城镇居民医保财政补助标准提高到360元，在全市范围内推开大病保险，对新农合住院患者吃中药（目录内）全额报销。新增50所精品幸福院、30个居家养老服务中心、1000个“健康小屋”。开工改造保障性安居工程14950套（户）、基本建成26000套（户），改造农村危房4200户。实施农村饮水安全提升工程，优先改善西部山区115个村、9.5万人的饮水条件。加大扶贫攻坚力度，确保10万人稳定脱贫。

统筹发展社会事业。优先发展教育事业，实施第二期学前教育三年行动计划，推进义务教育优质均衡发展，建成50所农村标准化中小学，搞好主城区住宅区配套中小学、幼儿园建设，开工建设西部省级示范性高中，加快现代职业教育集团实质性运作，提升高等教育办学水平。提高医疗保障水平，加快市儿童医院、市精神病医院项目建设。弘扬社会主义核心价值观，加快构建现代公共文化服务体系，加强文物和历史文化名城名镇名村保护。推进全民健身活动，办好市第十二届运动会。持续稳定低生育水平。

全面创新社会治理。完善社会稳定风险评估机制，扎实做好信访工作。健全公共安全应急机制，提高突发事件应急处置能力。加强民间融资管理，依法打击和妥善处置非法集资，全面防范金融风险。认真贯彻新《安全生产法》，严格落实“党政同责、一岗双责”，严防重特大安全生产事故发生。狠抓食品药品安全监管，确保全市人民饮食用药安全。充分发挥“三网”防控作用，依法打击各类犯罪活动，努力创造和谐稳定的社会治安环境。

加强国防教育和国防后备力量建设，争创全国双拥模范城“八连冠”。统筹做好新闻出版广电、气象防震减灾、民族宗教、邮政通信、人民防空、妇女儿童、老龄、残疾人等各项工作。同时，认真编制好“十三五”规划，引领全市经济社会更好更快发展。

各位代表！面对繁重的发展任务，我们一定戮力同心，真抓实干，尽责有为，以经济社会发展的新成效，回报全市人民的信任和重托！

三、全面加强法治政府建设

积极适应法治建设新要求，确立依法行政新思维，坚决维护宪法法律权威，把法治思维和法治方式贯穿政府工作全过程。

（一）依法明责定权，转变政府职能。坚持法定职责必须为、法无授权不可为，把政府职能切实转向经济调节、公共服务、市场监管、社会管理和环境保护，坚决克服懒政、怠政，坚决惩处失职、渎职。建立健全权力清单、责任清单和监管清单制度，年内制定市本级“监管清单”和“责任清单”。大力提高办事效率，推进网上服务，实现行政审批事项网上一体化办理和联合审批。

（二）依法科学决策，坚持执政为民。坚持把公众参与、专家论证、风险评估、合法性审查、集体讨论决定作为行政决策法定程序，认真落实政府法律顾问制度，做到决策科学、程序正当、过程公开、责任明确。推行重大决策终身责任追究制度及责任倒查机制，对决策失误造成重大损失、恶劣影响的，依法予以追责。

（三）依法接受监督，规范权力运行。自觉接受人大的法律监督、工作监督和政协的民主监督，虚心接受社会监督和舆论监督。加强执法监督，严格公正文明执法。强化和完善各级政府内部层级监督机制，对政府采购、政府投资、公共资源转让、国有资产监管等权力集中的部门和岗位，年内一律实行分事行权、分岗设权、分级授权和定期轮岗。坚持以公开为常态、不公开为例外原则，全面推进政务决策公开、执行公开、管理公开、服务公开、结果公开，重点推进财政预算、公共资源配置、重大建设项目批准和实施、社会公益事业建设等领域的政府信息公开，真正让行政权力在阳光下运行。

（四）依法从严治政，保持优良作风。巩固和深化党的群众路线教育实践活动成果，深入贯彻落实中央“八项规定”精神和国务院“约法三章”，坚决纠正“四风”问题。加强党风廉政建设，严格落实“一岗双责”，强化纠风和专项治理，坚决查处权力寻租、权钱交易和损害群众利益的行为，以反腐倡廉的实效保障发展、取信于民。坚持从严从俭，严格控制一般性支出，做到“三公”经费只减不增，确保把有限的财政资金用到最有效率、最急需的地方。

各位代表！创新发展形势催人，转型升级责任重大，绿色崛起时不我待。让我们在省委、省政府和中共邯郸市委的正确领导下，坚定信心，振奋精神，鼓足干劲，锐意进取，为早日建成宜居宜业宜游的富强邯郸、美丽邯郸而努力奋斗！
